# Beyond fossil calibrations: realities of molecular clock practices in evolutionary biology

**DOI:** 10.3389/fgene.2014.00138

**Published:** 2014-05-26

**Authors:** Christy A. Hipsley, Johannes Müller

**Affiliations:** ^1^Museum für Naturkunde, Leibniz-Institut für Evolutions- und BiodiversitätsforschungBerlin, Germany; ^2^Berlin-Brandenburg Institute of Avanced Biodiversity ResearchBerlin, Germany

**Keywords:** molecular clock, divergence dating, calibration, fossil, vertebrate paleontology, node age prior

## Abstract

Molecular-based divergence dating methods, or molecular clocks, are the primary neontological tool for estimating the temporal origins of clades. While the appropriate use of vertebrate fossils as external clock calibrations has stimulated heated discussions in the paleontological community, less attention has been given to the quality and implementation of other calibration types. In lieu of appropriate fossils, many studies rely on alternative sources of age constraints based on geological events, substitution rates and heterochronous sampling, as well as dates secondarily derived from previous analyses. To illustrate the breadth and frequency of calibration types currently employed, we conducted a literature survey of over 600 articles published from 2007 to 2013. Over half of all analyses implemented one or more fossil dates as constraints, followed by geological events and secondary calibrations (15% each). Vertebrate taxa were subjects in nearly half of all studies, while invertebrates and plants together accounted for 43%, followed by viruses, protists and fungi (3% each). Current patterns in calibration practices were disproportionate to the number of discussions on their proper use, particularly regarding plants and secondarily derived dates, which are both relatively neglected in methodological evaluations. Based on our survey, we provide a comprehensive overview of the latest approaches in clock calibration, and outline strengths and weaknesses associated with each. This critique should serve as a call to action for researchers across multiple communities, particularly those working on clades for which fossil records are poor, to develop their own guidelines regarding selection and implementation of alternative calibration types. This issue is particularly relevant now, as time-calibrated phylogenies are used for more than dating evolutionary origins, but often serve as the backbone of investigations into biogeography, diversity dynamics and rates of phenotypic evolution.

## Introduction

Divergence dates estimated from molecular phylogenies provide critical information on the timing of historical evolutionary events, including the temporal origins of clades. These dates are now integrated into a wide array of biological investigations, including studies of ancient dispersal mechanisms, adaptive radiations and species interactions. Despite major advances in phylogenetic methods (e.g., variable rate models; Drummond et al., 2006, Bayesian inference; Drummond and Rambaut, 2007, data partitioning; Nylander et al., 2004), calibration of the molecular clock continues to be the most significant source of variation among estimated dates (Marjanović and Laurin, 2007; Ho and Phillips, 2009; Inoue et al., 2010; Sauquet et al., 2012). Therefore, explicit justification and proper implementation of clock calibrations are essential to ensuring accurate reconstructions of the evolutionary past.

In response to these requirements, we the authors participated in a BioSynC Synthesis meeting in 2009 on the appropriate use (and misuse) of fossil calibrations, ultimately leading to a publication in *Systematic Biology* titled, “Best practices for justifying fossil calibrations” (Parham et al., 2012). This article, co-written by 25 paleontologists and molecular biologists, outlines a rigorous protocol for selecting and reporting fossil-based calibrations. Although the study mainly focuses on the vertebrate fossil record and its major divergences (e.g., crocodile-bird, human-chimpanzee), it also became apparent during our discussions that many workers dating molecular phylogenies rely on external calibrations other than fossil dates. This is likely because many soft-bodied organisms, including some invertebrates, plants and fungi, leave little to no fossil evidence of their ancient existences, making it impossible to implement specimen-based calibrations in reconstructions of their temporal pasts.

In lieu of appropriate fossils, many workers instead rely on geological events, substitution rates, known sampling dates, or secondarily and even tertiarily derived node ages to calibrate the molecular clock. While each of these calibration types has its strengths and weaknesses, the attention they have garnered in the scientific community seems small compared to the large number of commentaries, reviews and databases dedicated to the use of fossil calibrations (see Parham et al., 2012 and references therein). This imbalance of dialogue between researchers using alternative (non-fossil-based) calibrations and those focusing on paleontological material is not only detrimental to researchers wishing to reliably date their clades of interest, but also to the results of many studies relying on divergence estimates as a backbone for independent analyses of, among other things, diversification dynamics, biogeography, rates of phenotypic evolution, and character correlation.

The purpose of the present study is therefore to review current patterns in calibration types employed within the major taxonomic groups (e.g., vertebrates, invertebrates, plants, fungi, bacteria) and compare this to the number of publications discussing their appropriate use. Our goal is to identify and draw attention to areas of molecular dating research deserving of increased attention, with the hope that their respective workers will formulate a consensus on the best practices regarding choice and implementation of alternative calibration types.

## Methods

Current patterns in calibration use were assessed by a survey of relevant literature published in the past seven years. Using the Web of Science database (http://webofknowledge.com/), we searched topic terms [(molecular clock^*^ or divergence dat^*^) and calibrat^*^)] from 2007 to 2013 (2007 marking the release of the Bayesian dating software BEAST; Drummond and Rambaut, 2007). For inclusion in our survey, each paper was required to include an original phylogenetic analysis based on molecular data (but could also integrate morphological characters in a total evidence approach) and include an ultrametric time-calibrated tree (or table or figure) providing node age estimates. The focal taxonomic group of each analysis was recorded, as well as calibration type (see below). For single papers analyzing multiple independent data sets (e.g., parasite and host phylogenies) or the same data set with different calibration types, each analysis was scored separately.

Calibrations were categorized as: (1) *Fossil*. The earliest known fossil assigned to a lineage provides a minimum age constraint on the divergence event (i.e., internal node) at the base of its clade (Donoghue and Benton, 2007). Depending on the quality of the fossil record, the probability that the actual divergence falls around the fossil date may be expressed as a parametric distribution between minimum and maximum bounds (i.e., soft bounds; Yang and Rannala, 2006). (2) *Geological event*. Geological calibrations are assigned to internal nodes based on the assumption that phylogenetic divergence was caused by vicariance. Examples include the appearance of land bridges generating barriers to gene flow in aquatic organisms (minimum age constraint), or the emergence of an island on which a clade is inferred to have diversified (maximum age constraint) (Ho et al., 2011). As with fossils, the degree of uncertainty surrounding correspondence between the geological event and date of divergence may be expressed probabilistically. (3) *Sampling date*. Data sets containing sequences isolated at different times, i.e., heterochronous data, are calibrated by assigning known sample ages to terminal nodes in the phylogeny. Temporal information is based on the date of sequence isolation for rapidly evolving organisms like viruses and bacteria, or on radiocarbon dating of preserved material from which ancient DNA is extracted (Shapiro et al., 2011). For serially sampled sequences, node ages are treated as exact, i.e., point calibrations, while radiocarbon dates provide minimum age constraints with some degree of uncertainty (Ho and Phillips, 2009). (4) *Substitution rate*. In the absence of external calibrations, a known substitution rate may be applied to sequence data to convert genetic distance into time. This rate can be estimated by direct observation of genetic change, provided that the temporal range over which sequences are sampled is large relative to the rate of mutation (Drummond et al., 2003). Substitution rates may also be calculated indirectly from dated molecular phylogenies, in which case rate estimates depend on the calibration(s) applied in the original study (Ho and Phillips, 2009). (5) *Secondary calibration*. Secondary calibrations are node ages derived from previous analyses, applied to an independent data set without reference to the original calibration(s) used to generate them (Shaul and Graur, 2002). This category was also reserved for studies citing a specific calibration date but no source.

To evaluate patterns in discussions of proper calibration use, we identified papers from our search considered “review-like” and scored them for taxonomic group and calibration type using the above categories. Review-like papers should be general in taxonomic scope, i.e., above the family level, and have selection and implementation of clock calibrations as their main focus. They are not required to present a phylogenetic tree, although they may include examples based on simulated or empirical data. Papers focusing on the setting of parametric distributions describing calibration uncertainty (e.g., Heath, 2012; Warnock et al., 2012) were included in the main survey. As above, review-like papers discussing multiple calibration types or groups were scored separately for each. Data were summarized in JMP® 10 (Cary, NC: SAS Institute Inc.) and visualized in Microsoft Excel 2011. A list of all literature included in the survey is available in Supplementary Material.

## Results

**Figure 1:**
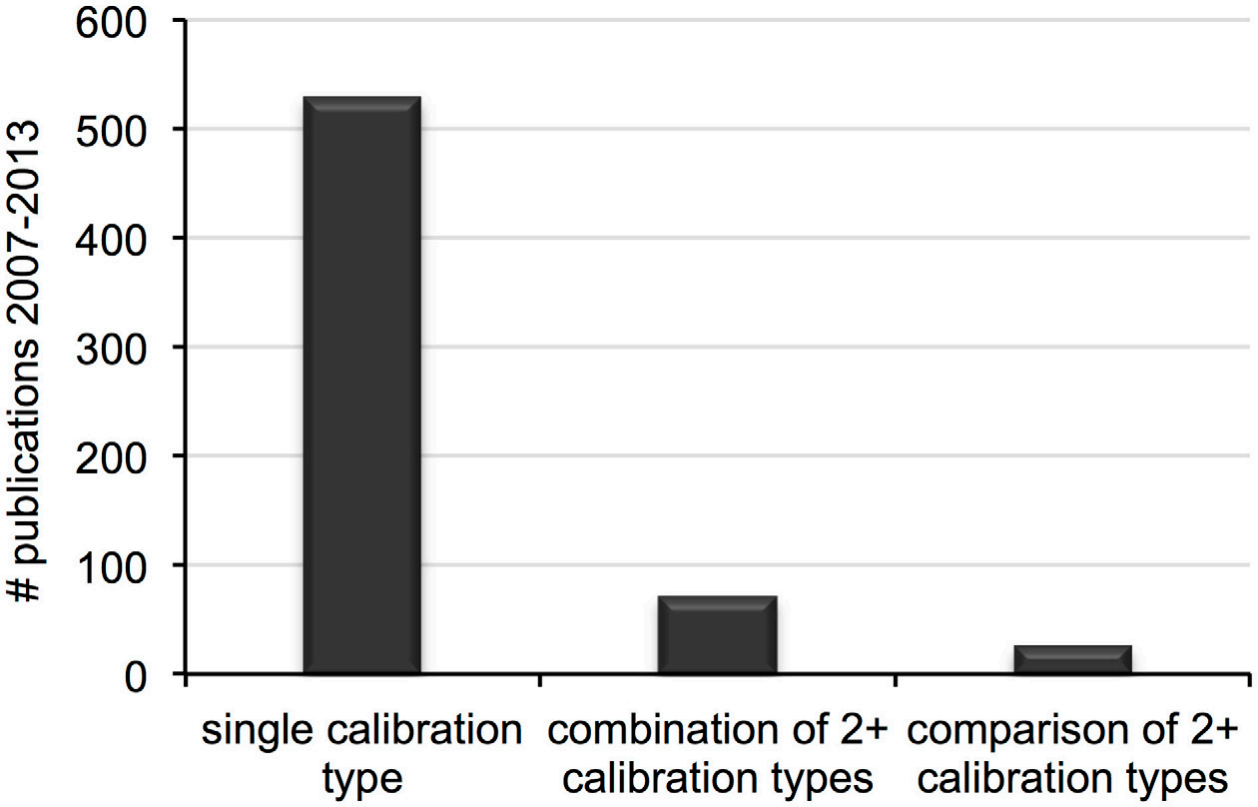
**Number of distinct calibration types (e.g., fossil, geological event) used in single divergence dating analyses published between 2007 and 2013**.

Our initial search resulted in 798 records. After controlling for quality according to the conditions above, 613 unique publications were available for survey. Of those, 562 were considered for clock calibration, the majority of which included a single analysis with one calibration type (e.g., fossil, geological event). The remaining 97 publications included multiple analyses, either on independent data sets or on the same data set using combinations or comparisons of two (or more) calibration types (Figure 1). After accounting for publications with multiple investigations, a total of 697 individual analyses were scored.

**Figure 2:**
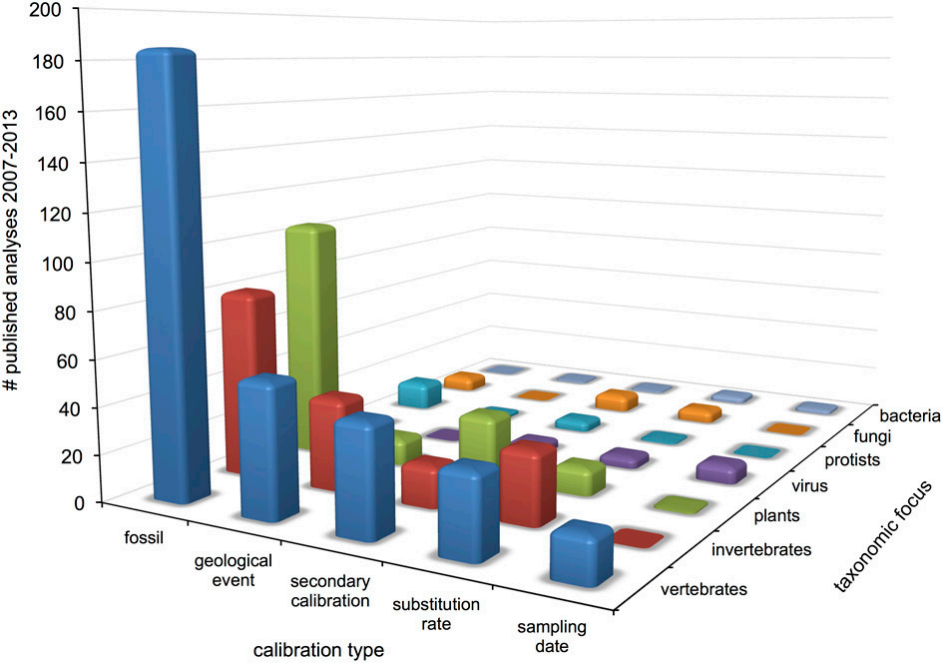
**Patterns of molecular clock calibration types applied among major taxonomic groups from 2007 to 2013**.

**Figure 3:**
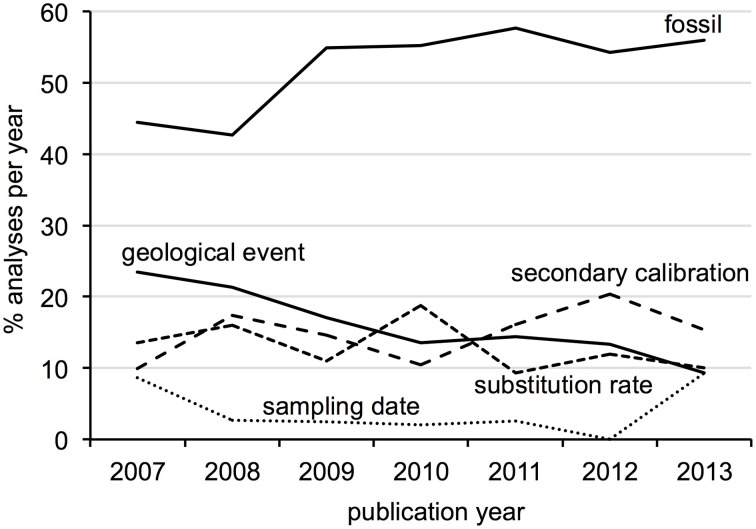
**Trends in calibration use for each type as percent of the total analyses published per year**.

Surveyed patterns in calibration use are summarized in Figure 2. Fossil calibrations were implemented in just over half of all analyses (52%), followed by geological events and secondary calibrations (15% each), substitution rate (12%) and sampling date (4%). Five analyses used anthropological events as external calibrations, which were considered unique from the other types. The majority of all analyses (70%) focused on metazoan groups, of which twice as many were vertebrate than invertebrate. Plants accounted for 21% of analyses, followed by viruses, protists, fungi (roughly 3% each) and bacteria (1%). Within Metazoa, arthropods were the most commonly studied group, while molluscs and other invertebrates like annelids, sponges and jellyfish were the least. Mammals were the most commonly investigated vertebrates, followed by fish, reptiles, birds and amphibians in decreasing order. Trend in calibration use also vary by year (Figure 3), with fossil and secondary calibrations showing a relative increase over time, while geological events and substitution rates show a slight decrease. Sampling date, the least used of all calibrations, remains low from 2008 to 2012, but increases from 0 to 9% in the last year.

**Figure 4:**
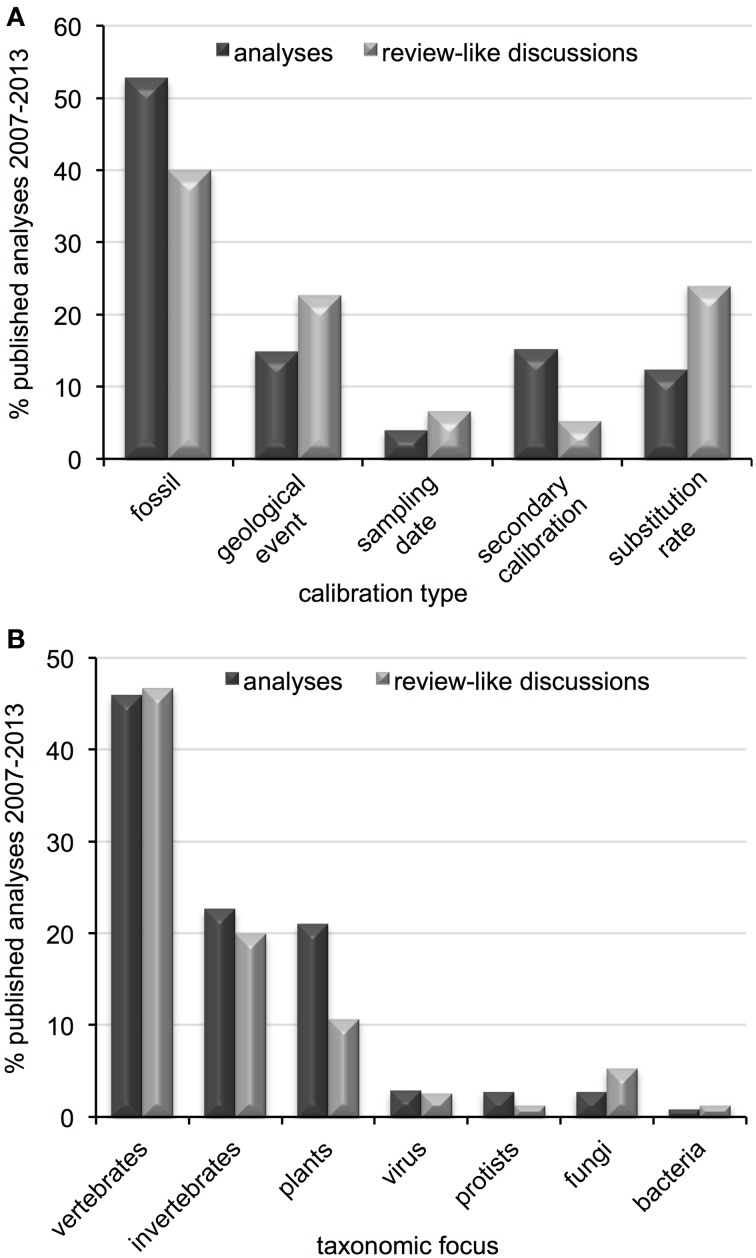
**Comparison of patterns in molecular clock analyses and review-like discussions regarding (A) calibration type and (B) taxonomic focus, published between 2007 and 2013**.

**Figure 5:**
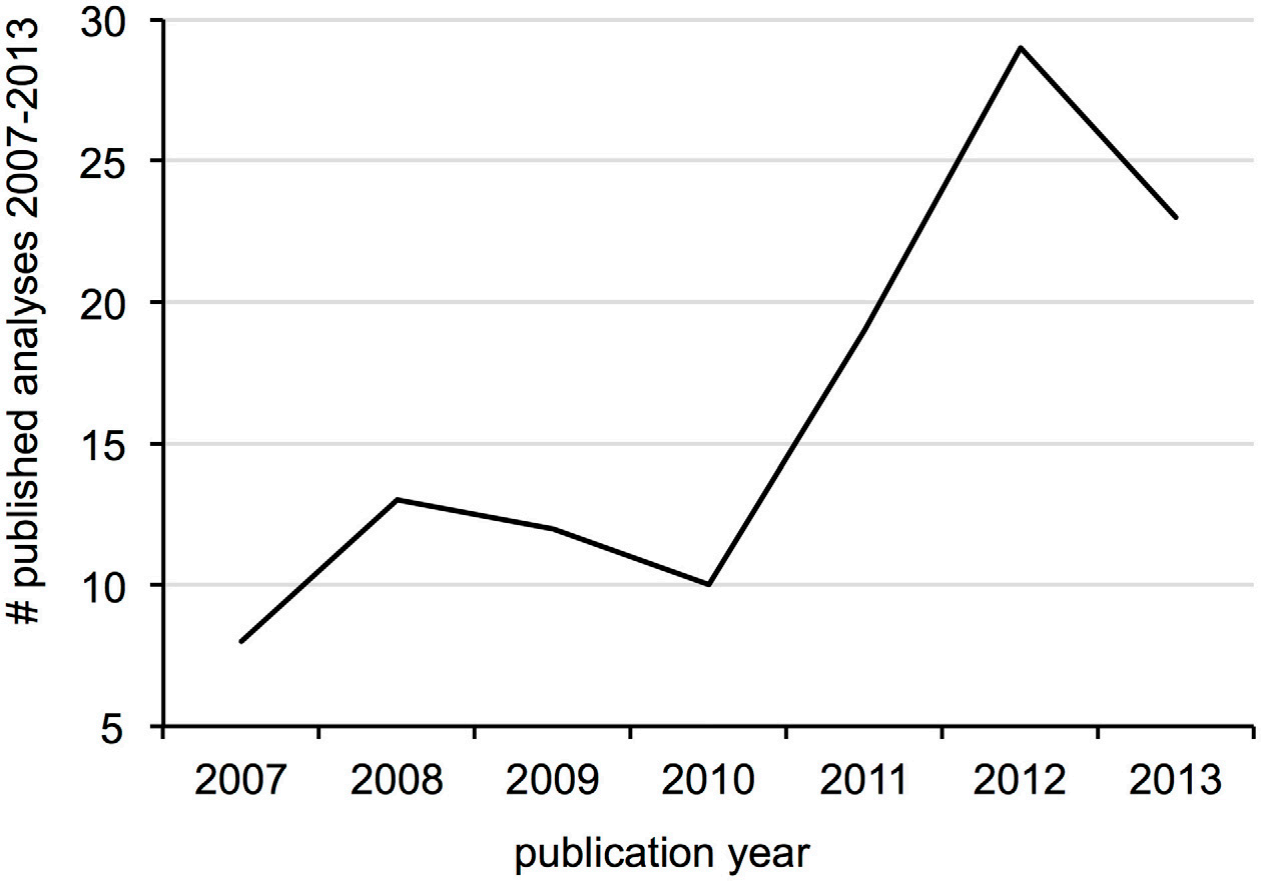
**Number of analyses published per year using secondarily derived dates as molecular clock calibrations, showing a recent upward trend**.

Fifty-one publications from the past seven years were considered review-like, as they concentrated primarily on the selection and implementation of calibration data in divergence dating analyses. The majority of discussions concentrated on fossil calibrations, with relatively few mentions of secondary calibrations or sampling dates (Table 1). Just over one-third of discussions were general in taxonomic scope, although the majority of those citing empirical data used vertebrate examples. Of those with taxonomic foci or examples, most concentrated on metazoans (50% vertebrates, 20% invertebrates), 11% on plants, 6% on fungi and less than 3% each on viruses, protists and bacteria (Table 1). When comparing the subjects of these discussions to current divergence dating practices, some disproportion is observed regarding calibration type (Figure 4A) and taxonomic group (Figure 4B), particularly for plants and secondary calibrations which are both underrepresented in methodological evaluations. This imbalance is most dramatic for the latter, as the use of secondary calibrations has increased dramatically since 2007 (Figure 5), independent of the yearly increase in numbers of divergence dating studies in general (Figure 3).

**Table 1:** **Summary of subjects of review-like articles published from 2007 to 2013 on the proper use of molecular clock calibrations**.

**Calibration type focus**	**# discussions**
Fossil	29
Geological event	16
Sampling date	5
Secondary calibration	3
Substitution rate	16
**Taxonomic group focus**	**# discussions**
General	25 (18 examples: 10 vertebrate, 3 invertebrate, 5 plant)
Vertebrate	25
Invertebrate	11
Plant	3
Virus	2
Protist	1
Fungus	4
Bacteria	1

## Discussion

Our survey shows that results from divergence dating analyses are now incorporated into an astounding array of biological and geological investigations (Table 2), making the accuracy and precision of divergence estimates of paramount importance to our understanding of evolutionary history. Calibration of the molecular clock has been shown to be the most significant factor influencing divergence dates (Inoue et al., 2010; Sauquet et al., 2012), such that choice and implementation of clock calibrations should constitute a central dialogue in evolutionary biology. Indeed, the high number of citations for many review-like papers on the subject as well as the high impacts of the journals in which they are published indicates a large audience for discussions on proper calibration use. We found that although some taxonomic groups and calibration types were neglected in these discussions (e.g., plants, secondary calibrations), others like substitution rates and geological events were overrepresented, leading to an overall disproportion between calibration implementation and guidelines on their proper use. This is particularly alarming regarding the application of secondarily derived dates as node age priors (see discussion below), a practice that has more than tripled in the past seven years (Figure 5).

**Table 2:** **Examples of applications of molecularly-derived divergence dates**.

**Investigations of:**	**Focus**	**References**
Phylogeography	Lichenized fungi ancestral ranges	Amo de Paz et al., 2012
Dispersal mechanisms	Transatlantic rafting by rodents; island-hopping reptiles; long-distance plant dispersal	Rowe et al., 2010; Davy et al., 2011; Nylinder et al., 2012
Adaptive radiations	Repeated colonization and isolation of Hawaiian honeycreepers; lack of replicated adaptive radiations in Caribbean snakes	Lerner et al., 2011; Burbrink et al., 2012
Diversification drivers	Marine hotspots of reef-associated fish	Alfaro et al., 2007
Geological events	Peruvian Andes uplift (nematodes); emergence of New Caledonia (fig tree/wasps)	Picard et al., 2008; Cruaud et al., 2012
Species associations	Acacia plants/ants mutualism; blood parasites/bats movements; host-switching of mammalian sucking lice	Gomez-Acevedo et al., 2010; Light et al., 2010; Hamilton et al., 2012
Cryptic diversity	Species status of African forest duikers	Johnston and Anthony, 2012
Speciation mechanisms	Accumulation of reproductive incompatibility in cichlid fish and waterfowl; self-sterility in flowering plants	Gonzalez et al., 2009; Stelkens et al., 2010; Ferrer and Good, 2012
Key innovations	Antifreeze glycoproteins in Antarctic fishes; fleshy fruit; shift to xeric habitats in legumes	Egan and Crandall, 2008; Biffin et al., 2010; Near et al., 2012
Trophic novelties	Multiple origins of novel feeding modes in reef fish	Cowman et al., 2009
Conservation	Genetic endemism of threatened cloud forest biota	Francisco Ornelas et al., 2013
Paleoecology	Dinosaurs as cycad dispersal agents; amphibious ancestry of echidnas	Phillips et al., 2009; Nagalingum et al., 2011
Convergent evolution	Body plans of skates and rays; C4-specific enzymes in sedges	Besnard et al., 2009; Aschliman et al., 2012
Diversity dynamics	Museum vs. evolutionary cradle models in butterflies	Condamine et al., 2012
Chromosomal evolution	Karyotype origination in rodents	Castiglia et al., 2012
Mass extinctions	Rise of mammals at the K-Pg boundary	Meredith et al., 2011; Springer et al., 2012

Based on our literature survey, below we summarize recent patterns in clock calibration use, and discuss potential pitfalls associated with each methodology. Although our list is not exhaustive, we attempt to provide a reasonable overview of calibration implementation, while focusing on progress made in the past seven years. Our goal is not to single out any individual study or author for criticism, but to draw attention to areas of dating research deserving of greater attention. We finally stress that divergence dating is a dynamic science, and that a realistic approach to dating one's clade of interest may involve a (possibly suboptimal) combination of the methods described below.

### Fossils

Treatment of paleontological calibrations has been discussed extensively in recent years (e.g., Benton and Donoghue, 2007; Donoghue and Benton, 2007; Rutschmann et al., 2007; Gandolfo et al., 2008; Lee and Skinner, 2011; Parham et al., 2012), with uncertainty in fossil age and phylogenetic position still presenting the two greatest challenges. Incorporation of temporal uncertainty into dating analyses is now common practice (Heled and Drummond, 2012), although parameterization of node age priors is often arbitrary or idiosyncratic at best. Fortunately, recent methods have been developed for the objective quantification of prior distributions based on stratigraphic occurrence and preservation rates of focal taxa (Dornburg et al., 2011; Wilkinson et al., 2011; Nowak et al., 2013; Sterli et al., 2013), signifying a promising step toward biologically relevant constraints. Dangers of phylogenetic misplacement have also stimulated novel approaches, in which molecular and morphological data are combined to assess uncertainty in fossil position, which is then used to determine confidence intervals surrounding dates derived from those calibrations (Lee et al., 2009). A superior alternative may be to treat fossils as non-contemporaneous terminal taxa, thus allowing direct assignment of ages to fossil tips (Pyron, 2011; Ronquist et al., 2012; Wood et al., 2013). Both empirical and theoretical work shows that the addition of fossil taxa can improve branch length estimation and phylogenetic support (Wiens, 2009; Pyron, 2011), suggesting that combined evidence analyses may supplant purely molecular frameworks in the near future.

Other advances regarding paleontological calibrations involve selection of the fossils themselves, either through single-fossil cross-validation (Near et al., 2005), fossil coverage methods (Marshall, 2008), or Bayesian multi-calibration techniques (Sanders and Lee, 2007). These apply not only to fossilized hard parts (e.g., shells, bones, wood), but also to ichnofossils recording biological activity. Ancient feeding tracks (Gomez–Zurita et al., 2007), coprolites (Zhong et al., 2009), and fossilized termite mounds (Brandl et al., 2007) have all been used to date lineages for which body fossils are lacking, and the first appearance of taxon-specific biomarkers has served to constrain the emergence of eukaryotes (Peterson et al., 2008). Amber inclusions, formed when tissue is trapped in fossilized resin, constitute another important source of paleontological constraints, particularly for small and soft-bodied organisms such as insects (Wilson and Pitts, 2010; Kuntner et al., 2013), plants (Feldberg et al., 2013) and fungi (Amo de Paz et al., 2012). It should be noted, however, that the ages of some amber remains controversial (e.g., Dominican amber; Pitts et al., 2010), such that divergences constructed around those dates should be treated with care. One common instance where fossil calibration may not be possible is in analyses of shallow divergences (e.g., intrageneric, phylogeographic studies), since even for groups with fossil representatives, the lack of variability in diagnostic hard parts at lower taxonomic levels means that fossils cannot be placed confidently within genera.

In cases where paleontological evidence of the focal group is completely lacking, some authors resort to adding external branches to the phylogeny in order to accommodate distant yet potentially inappropriate fossil calibrations. Particularly for groups whose biological properties (e.g., mutation rate, generation time) differ significantly from their relatives, distant external calibrations are more likely to bias divergence estimates than accurately reflect evolutionary history (Cutter, 2008). Relaxed molecular clock models that accommodate rate variation may help to minimize this risk, and for rapidly evolving species like *Drosophila*, direct estimates of mutation rates from laboratory populations have been used to infer divergences at deeper nodes (Cutter, 2008; but see substitution rates discussion below). Alternatively, when no appropriate constraints are available, uncalibrated molecular clocks provide relative clade ages that can support or reject temporal congruence of historical evolutionary events, such as parallel distributions (Loader et al., 2007) and symbiotic interactions (Hibbett and Matheny, 2009). However, caution should still be taken when using this approach, as rates of molecular evolution tend to vary among clades. Therefore, tests of significant rate variation between focal groups, for example using relative rate tests, should be performed prior to the application of a global molecular clock.

Lastly, one should keep in mind that paleontology is an ever-expanding field with new material and technologies constantly emerging, albeit at a slower rate than advances in molecular biology (Sterli et al., 2013). Not only is our ability to extract information from the fossil record improving, new interpretations of stratigraphic and character-based data through sampling standardization, stable isotopes, geomagnetic polarity and X-ray computed tomography provide increasingly detailed and well-placed constraints on a wide array of taxa. Dissemination of this knowledge through public directories such as the Paleobiology Database (www.paleobiodb.org, see also www.fossilworks.org) and Date-a-Clade (www.fossilrecord.net/dateaclade) make it easier than ever for non-paleontologists to access up-to-date calibration data. In addition to paleontological databases, time-calibrated supertrees, or timetrees (e.g., Marjanović and Laurin, 2007, 2013), have been produced specifically to provide minimum (and sometimes maximum) divergence dates based on the fossil record. In some cases this information has prompted revision of previously applied calibrations (e.g., some of the earliest known bilaterians; Dong et al., 2008, lepidopterans; de Jong, 2007), while in others recent fossil findings have led to greater congruence of molecular and paleontological estimates (e.g., placental mammals; Goswami, 2012). As fossils represent our only hard evidence of the evolutionary past, these trends indicate a still rich and vital role for paleontologists and paleontological material in dating the tree of life.

### Geological events

Despite the prevalence of geological calibrations in recent studies, the majority of review-like evaluations caution against their use based on the unfounded assumption of vicariance (de Jong, 2007; Ho, 2007; Forest, 2009; Wilke et al., 2009; Goswami and Upchurch, 2010; Kodandaramaiah, 2011; Cohen, 2012; Pirie and Doyle, 2012; Mayr, 2013). Long considered to be the predominant mode of speciation, vicariance is often invoked to explain disjunct distributions of related taxa, such as those with presumed Gondwanan affinities. However, a growing number of studies indicate that transoceanic dispersals, even in groups assumed to have low mobility like plants (Knapp et al., 2005), amphibians (Vences et al., 2003) and burrowing reptiles (Vidal et al., 2008), are more common than previously thought. In cases where dispersal postdates the presumed biogeographic event, the assumption of vicariance will result in spuriously old divergence estimates (Kodandaramaiah, 2011). In contrast, some clades may be older than their inferred geographic isolation (e.g., the tuatara lineage predates separation of New Zealand from Gondwana; Jones et al., 2009, 2013, Galapagos giant tortoises separated from their mainland relatives before the emergence of the oldest Galapagos island; Parent et al., 2008), in which case the geological calibration will underestimate the actual divergence date.

Unfortunately, many studies continue to use ages derived from geological calibrations to support biogeographic hypotheses, falling into a trap of circular reasoning by presupposing the very speciation mode they are trying to test. It such cases, it is recommended that geological calibrations be either avoided completely (Kodandaramaiah, 2011) or be assessed independently of biological (e.g., paleontological) information (Waters and Craw, 2006). When appropriate fossils are unavailable, as may already the case when geological calibrations are employed, secondary calibrations derived from previous analyses provide another alternative (but with its own associated pitfalls, see below). It should be noted, however, that the assumption of vicariance may be more justified in certain groups than others (e.g., flying animals may cross marine barriers more easily than salt-intolerant ones), although guidelines by which to apply such *a priori* hypotheses to calibration modes are still lacking.

Another fundamental concern surrounding geological calibrations is uncertainty in the sequence and timing of the geological events themselves, as continental drift and the formation of barriers can occur over millions of years (Upchurch, 2008). Multiple paleogeographic models exist for the breakup of Gondwana during the Jurassic and Cretaceous (Upchurch, 2008), and the timing of younger events like the rise of the Panamanian Isthmus is far from precise (Kodandaramaiah, 2011). Despite substantial margins around tectonic dates, many studies treat geological calibrations as exact, when in fact they are typically more poorly constrained than fossil ages (Goswami and Upchurch, 2010). Furthermore, the fossil record indicates that speciation and extinction events associated with the formation of barriers are often more gradual rather than abrupt (Marko, 2002), such that divergence and geological events may be uncorrelated over time (Papadopoulou et al., 2010). For example, the final closure of the Isthmus of Panama at 3.5 million years ago is one of the most commonly employed calibration dates for marine taxa (Lessios, 2008), although geminate species pairs on either side likely diverged well before seaway constriction (Marko, 2002).

In reality, some geological constraints may be more accurate than others, depending on the environmental context of the evolutionary events that they calibrate. For example, if the estimated topology of a clade inhabiting an island archipelago matches the sequence in which the islands emerged without evidence of multiple colonizations, it may be reasonable to use island ages as maximum constraints (e.g., Cox et al., 2010). At the same time, caution should be exerted in such superficially simple situations, as the use of maximum island ages as lower bounds can cause severe overestimates for organisms inhabiting ancient archipelagos like Hawaii (Obbard et al., 2012). Other examples in which geological dates may be appropriate are rapid events like river captures and reversals, which can be directly related to allopatric speciation and therefore represent precise spatiotemporal disruptions leaving testable genetic signatures (Waters et al., 2007; Burridge et al., 2008).

As with the fossil record, new biogeographical information is constantly emerging making previously applied dates significantly altered or obsolete. For example, genetic barcoding of African trypanosomes revealed recent New World dispersal, invalidating the continental separation event used to calibrate their origins (Hamilton et al., 2009). Similarly, recent drill cores from Lake Malawi showed relative ecological stability over the past 70,000 years, calling into question divergence estimates tying Malawi cichlid radiations to habitat fluctuations since the Last Glacial Maximum (Cohen, 2012). Advances in growth models and geological methods also indicate older emergences of some Hawaiian islands than previously thought, meaning that studies relying on prior dates will tend to underestimate clade ages (Obbard et al., 2012).

Given the many limitations of geological calibrations, we generally advocate alternative methods of clock calibration, although we acknowledge that for some taxa a geological date, at least at first sight, is the only available option. In such cases, one should attempt to perform independent assessments using other sources of evolutionary information such as substitution rates or related fossils, and ideally apply multiple probabilistic priors to assess alternative geographical scenarios (e.g., Mello and Schrago, 2012). Other potential options include the use of geological calibrations from outside of the focal clade's current distribution, or a “reverse” approach in which divergence is constrained to be older than the postulated biogeographic event, in order to examine compatibility between dates obtained for deeper nodes and other (older) paleogeographical disjunctions. For example, Nattier et al. (2011) constrained the origin of New Caledonian crickets to predate the most recent island re-emergence, resulting in a divergence estimate 47 million years older than the closest corresponding biogeographic event. Based on this discrepancy, they were able to reject an island-hopping scenario that would have allowed New Caledonian species to persist during submersion, in favor of more recent colonization events supported by the fossil record and studies of independent groups.

### Substitution rates

As opposed to fossil and geological calibrations, the primary concern regarding substitution rates in divergence dating is the time scale over which those rates are measured. Rates of molecular change observed between generations of laboratory and pedigree lines have been shown to far exceed those inferred from the fossil record, resulting in a phenomenon of time dependency that is still under heavy debate (Ho et al., 2011). Some authors argue that elevated rates observed over recent timescales reflect the spontaneous rate of (non-lethal) mutations, while older timeframes yield the long-term substitution rates seen in phylogenetic data. Substitution rates are generally expected to be lower than rates of mutation, since natural selection tends to remove deleterious mutations over time. Therefore, distant relatives having undergone more opportunity for selection will appear to have lower molecular rates than closely related taxa, thus displaying time-dependency (Ho et al., 2011). Cutter (2008) attempted to correct for this bias by focusing on divergence at synonymous sites in laboratory populations of nematodes and *Drosophila*, in order to approximate a neutral process of evolution required to date deep divergences in those clades. Although this method overcomes the challenges of time-dependency and lack of fossil calibrations (Ho and Lo, 2013), rapid saturation of such sites may raise additional problems, as basal branch lengths can become artificially compressed, leading to overestimated divergence dates (Phillips, 2009)

For taxa lacking empirical data, many authors apply substitution rates derived from independently calibrated trees to convert genetic distance into time. Here again, the issue of time-dependency emerges, as rates of molecular evolution appear negatively correlated with the ages of the calibrations used to estimate them (Ho et al., 2011). However, the strength of this relationship varies across lineages, timescales, genes and location of the calibrated node itself (within species vs. among), making it difficult to simultaneously correct for all of these factors. The application of local molecular clocks to different branches of the tree offers a potential solution for distinguishing between short- and long-term rates (Yoder and Yang, 2000), and Ho et al. (2011) recommend performing separate analyses for population vs. species-level divergences. Alternatively, some studies suggest that substitution rates vary predictably as a function of time, in which case they may be mathematically modeled and applied to the phylogenetic tree as a whole (Ho et al., 2007; Rodrigo et al., 2008).

Fortunately as with the above calibration types, substitution rates are being constantly updated and refined, thanks to long-term serial isolates (Morelli et al., 2010), large-scale pedigrees (Sun et al., 2012) and whole-genome sequencing (Roach et al., 2010). This is particularly true for human molecular estimates, which are increasingly moving away from a reliance on the human-chimpanzee split (ranging from 4 to 8 million years ago, depending on the source; Bradley, 2008), and are instead measured directly in human germline studies (Sun et al., 2012). These revised rates are expected to provide a more accurate timescale for events in human history, including divergence from Neanderthals and migration out of Africa (Endicott et al., 2009). Lastly, the increasing ability to amplify DNA from fossilized tissue means that substitution rates may be estimated directly from ancient sequences (Ho et al., 2007), although care must be taken to avoid upward biases due to oversimplified demographic models (Navascues and Emerson, 2009).

### Sampling dates

Unlike substitution rates applied across the phylogenetic tree, calibrations associated with heterochronous data are assigned to tips of the tree only, represented by molecular sequences with known sampling dates. This approach is mainly limited by the age ranges of the sequences themselves, extending from months or years for viral and bacterial samples, to hundreds of thousands of years in the case of ancient DNA (Willerslev et al., 2007). Rapidly evolving sequences from laboratory species are commonly involved in investigations of recent evolutionary phenomena, such as HIV dynamics (Wertheim and Worobey, 2009) and smallpox epidemics (Li et al., 2007), while ancient DNA is used to estimate population- or species-level divergences and to correlate genetic variation with climatic change over time (Orlando et al., 2013).

As with fossil and geological calibrations, accuracy of sample ages plays a crucial role in determining the quality of divergence estimates derived from them (Molak et al., 2013). While some sampling dates are known with precision (e.g., through museum records or laboratory studies), others contain various sources of uncertainty. Precise radiometric dating is generally limited to <55k years (Ho et al., 2011), although error may be introduced through variation in decay rates, contamination, and conversion of radiocarbon years into absolute time (Molak et al., 2013). For older samples, dates are typically estimated indirectly from the stratigraphic layer in which they are found, leading to even greater error margins than direct dating methods (Ho and Phillips, 2009). Shapiro et al. (2011) presented guidelines for estimating unknown sampling dates by modeling age uncertainty at external nodes as parametric distributions in a Bayesian framework. Molak et al. (2013) extended this approach to show that the temporal distribution of samples had a greater impact on accurate divergence estimates than prior parameterization, with fewer older calibrations yielding more accurate dates than numerous younger ones.

Although sampling date is currently the least employed calibration type, we expect that future advances in radiometric dating and ancient DNA extraction, as well as the increasing ability to confidently incorporate sequences with contentious ages, will extend the application of this strategy to broader temporal and taxonomic scales. But there are again caveats to this approach. Differences in substitution rates of mitochondrial and nuclear genes mean that slowly evolving sequences most suited for inferring deep divergences (i.e., nuclear DNA) show little variability within the last millions of years, limiting the ability of direct (tip) calibrations. However, the combination of different marker types in divergence dating analyses may reveal complementary information about population dynamics at different timescales. Eytan and Hellberg (2010) used mitochondrial and nuclear sequence data in demographic reconstructions of Caribbean reef fish, recovering a complex history of recent and old population expansions that would have been otherwise obscured with a single marker type. Although this study was performed with a geological calibration, a similar multi-level approach could be taken using sampling dates for more recent phenomena, and an independent calibration type (e.g., fossil or geological event) at internal nodes for estimates of older evolutionary divergences.

### Secondary calibrations

Secondary calibrations represent the most commonly applied age constraint after fossils, despite an overwhelmingly negative attitude towards their use (e.g., Graur and Martin, 2004; Reisz and Müller, 2004; Ho, 2007; Hug and Roger, 2007; Forest, 2009; Pirie and Doyle, 2012; Sauquet, 2013). The main concern with this approach is that error associated with the primary calibration becomes subsumed into new estimates, resulting in divergence dates of increasingly dubious reliability. Many secondary dates derive from earlier studies using only a single calibration point (Graur and Martin, 2004), while others are extracted from analyses conducted at higher (and potentially distant) taxonomic scales (Pirie and Doyle, 2012). This latter approach may yield greater associated uncertainty if the focal taxa express different rates of molecular evolution than the originally calibrated clade. Furthermore, the nature of the original prior constraint (minimum vs. maximum) is typically ignored, thus affecting correct interpretation of primary estimates. In the worst case, compounded errors due to the disregard of uncertainty coupled with uncritical assessment of the original calibration(s) are propagated in subsequent analyses, resulting in divergence estimates with decreasing accuracy and thus diminishing scientific value.

However, we realize that for some clades, such as those in wet tropics lacking fossil records, no reliable means of clock calibration exists, making alternative calibration approaches void. Only then do we support the use of secondary dates, in which case special care must be taken to accurately report potential bias associated with the original study. When secondary calibrations are the last resort, a normally distributed prior may be useful for reflecting potential error in imported constraints, as uncertainty is expected to be equal on either side of the mean age (Ho, 2007; Forest, 2009). Alternatively, if errors (e.g., 95% highest posterior density) surrounding dates for a node are reported in the original publication, it may be appropriate to implement a similar error margin when applying that nodal age as a secondary calibration. When choosing the secondary calibration, it is also advisable to consult studies with multiple, justified (paleontological or otherwise) constraints, and to apply methodologies that account for rate variation and uncertainty in prior age. Lastly, one should explicitly report confidence intervals around the new dates, in order to allow others to properly assess their concordance with independent sources of evolutionary data as they come to light.

## Conclusions

The confounding nature of evolutionary rates and time in divergence dating analyses requires that the molecular clock be calibrated independently using information from the evolutionary timescale. Here we show that this evidence can come from multiple sources, ranging from mutation rates measured in pedigree and laboratory lines, to fossil material and geological events millions of years in the past. Although paleontological calibration is not always possible, age constraints based on other types of data provide alternative means that, when well justified, can contribute critical information on the evolutionary history of life. Given that several calibration methods are now available, discussions on proper implementation of clock calibrations should reflect current practices in their use. This refers not only to choice of calibration type, but also to the taxonomic group under study, for which the availability of different calibration types will vary. Particularly now, considering the increasing availability of molecular sequence data and programs for their analysis, the development of best practices regarding alternative calibration types will benefit not only the primary researchers implementing these methods, but also workers in complementary fields relying on well estimated dates for studies of independent phenomena, including biogeography, ancient dispersals, adaptive radiations and diversity dynamics.

### Conflict of interest statement

The authors declare that the research was conducted in the absence of any commercial or financial relationships that could be construed as a potential conflict of interest.
